# Varicose vein surgery after acute isolated superficial vein thrombosis in daily practice: INSIGHTS-SVT study

**DOI:** 10.1016/j.jvsv.2024.101917

**Published:** 2024-05-29

**Authors:** Thomas Noppeney, Eberhard Rabe, Ulrich Hoffmann, Alexandra Schimke, Andreas Heinken, Florian Langer, David Pittrow, Jens Klotsche, Horst E. Gerlach, Rupert Bauersachs, INSIGHTS-SVT study group, Christian Schnabl, Tina Winterbauer, Norbert Harriet Schön, Simone Werno, Georg Herman, Oliver Schmidt, Beate Dietrich, Martin Schünemann, Eberhard Rieker, Ulrich Ruppe, Gabriele Betzl, Thomas Noppeney, Peter Heilberger, Dimitrios Tsantilas, Andreas Köpp, Lutz Forkmann, Andreas Willeke, Gabriele Rothenbücher, Karl Förster, Jeanette Kießling, Gesche Junge, Ina Wittig, Dagmar Wilms, Christoph Schulte, Stephan Flüchter, Martina Kneist, Ulrike Kirsch, Thomas Herrmann, Alexandra Turowski, Karsten Hartmann, Wolfram Oettler, Heike Nelles, Jürgen Frank, Savvas Apostolidis, Dag-Alexander Keilhau, Renate Murena Schmidt, Iris Rocha Rivera-Reuver, Kerstin Augustin, Diethard Predel, Thomas Hertel, Ursula Schmeink, Simone Seibt, Jürgen Schreiner, Christine Zollmann, Eckart Möbius, Thomas Vollmer, Roswitha Brettschneider, Sabine Raulin, Siamak Pourhassan, Gerlind Läger, Robert Brandl, Rainer Schmiedel, Karoline Jager, Erika Mendoza, Jörg Schwuchow, Jan-Peter Siegers, Peter Gätzschmann, Dimitrios Zgouras, Werner Lang, Arne Clasing, Anatoli Ananin, Jörg Rutkowski, Christoph Kalka, Frank Ackermann, Fred Peter, Patricia Schaub, Jan Beyer-Westendorf, Bernadette Brado, Mario Schöniger, Sven Köpnick, Ferenc Biro, Birgit Linnemann

**Affiliations:** a Department for Vascular and Endovascular Surgery, University Hospital Regensburg, Regensburg, Germany; b Private Practice Dermatology and Phlebology, Bonn, Germany; c Division of Vascular Medicine, Medical Clinic and Policlinic IV, Ludwig-Maximilian University, Munich, Germany; d Medical Affairs, Mylan Germany GmbH, Bad Homburg, Germany; e Medical Affairs, Amgen GmbH, Munich, Germany; f II. Medical Clinic and Policlinic, Center for Oncology, University Medical Center Eppendorf, Hamburg, Germany; g Institute for Clinical Pharmacology, Medical Faculty, Technical University, Dresden, Germany; h Innovation Center Real-World Evidence, GWT-TUD GmbH, Dresden, Germany; i Epidemiology and Health Services Research, Deutsches Rheuma-Forschungszentrum Berlin, ein Institut der Leibniz-Gemeinschaft, Berlin, Germany; j Private Practice for Vascular Diseases, Viernheim, Germany; k Center for Thrombosis and Hemostasis, University Medical Center Mainz, Mainz, Germany; l Cardioangiologisches Centrum Bethanien CCB, Standort AGAPLESION Bethanien Krankenhaus, Frankfurt am Main, Germany

**Keywords:** Fondaparinux, Low-molecular weight heparin, Prognosis, Risk assessment, Superficial vein thrombosis, Surgical intervention, Venous thrombosis

## Abstract

**Objective:**

The aim of this study was to assess the utilization of surgical interventions in patients diagnosed with superficial vein thrombosis (SVT) and its potential association with the occurrence of venous thromboembolism (VTE) and bleeding events.

**Methods:**

INSIGHTS-SVT, a prospective, non-interventional, multicenter study in Germany, investigated the management and outcomes of patients with acute SVT who received conservative and/or invasive treatments at the discretion of the treating physician.

**Results:**

Among the 872 patients with 12-month data, 657 had medical therapy only, and 215 patients underwent vascular surgery (70 within 3 months of SVT diagnosis, 136 between months 4 and 12, and nine had an intervention in both periods). The most commonly performed procedures included endovenous thermal ablation, ligation of the saphenofemoral or saphenopopliteal junction, and vein stripping. The primary outcome of symptomatic VTE was observed in 5.8% of conservatively treated patients and 6.3% of those who underwent surgical intervention. Additionally, the secondary outcome of recurrent or extended SVT was documented in 4.7% of conservatively treated patients and 5.3% of invasively treated patients. Bleeding events occurred in 1.4% of conservatively treated patients and 2.1% of surgically treated patients. These differences were statistically not significant. Furthermore, our analysis indicated a potential protective effect associated with surgical treatments, such as ligation of the saphenofemoral or saphenopopliteal junction, stripping and endovenous thermal ablation, concerning the endpoint of VTE for patients when applied after 3 months from the index SVT event.

**Conclusions:**

In line with previous research, our study suggests that surgical interventions are not frequently employed in the management of SVT, although they may be warranted in select cases. Nevertheless, additional research is essential to gain a deeper understanding of the indications, criteria, and benefit of surgical interventions in the treatment of SVT.

**Article Highlights**

• **Type of Research:** Multicenter, prospective cohort study
• **Key Findings:** In 197 invasively and 675 conservatively treated patients with acute superficial vein thrombosis (SVT), symptomatic venous thromboembolism occurred in 6.3% vs 5.8%, recurrent or extended SVT in 5.3% vs 4.7%, and bleeding events in 2.1% vs 1.4% over 12 months.
• **Take Home Message:** Almost all patients in the study received anticoagulation alone (conservative treatment) or in combination with surgery. Surgery after SVT was rare. Patients treated invasively appeared to be “low-risk” compared with those treated conservatively, as they were younger and had fewer previous venous thromboembolism events, but had a higher rate of varicose veins in their medical history. There were no statistically significant differences between groups in outcomes at 12 months.

Acute superficial vein thrombosis (SVT) is a prevalent vascular condition characterized by the formation of blood clots in the superficial veins, mostly in the lower extremities. Although generally considered a benign condition, SVT is frequently associated with substantial pain, swelling, and discomfort in affected patients.^1^ Furthermore, if left untreated, SVT can progress to potentially life-threatening conditions such as deep vein thrombosis (DVT) or pulmonary embolism (PE).^2^ Therefore, prompt diagnosis and effective management of SVT are essential in averting these serious complications.

Recently, the European Society for Vascular Surgery (ESVS) issued its 2021 Clinical Practice Guidelines on the Management of Venous Thrombosis, providing evidence-based recommendations for the diagnosis, treatment, and prevention of venous thromboembolism (VTE), encompassing SVT.^3^ These guidelines advocate an SVT management approach largely contingent on symptom severity and the likelihood of progression to DVT or PE. Patients experiencing mild to moderate SVT may derive benefit from noninvasive interventions such as compression stockings and nonsteroidal anti-inflammatory drugs (NSAIDs).^4^ However, individuals with more extensive SVT or those facing a high risk of progression (eg, SVT close to saphenofemoral junction [SFJ], malignancy, extension of SVT clot) may necessitate more aggressive treatments, including anticoagulation or surgical intervention.

Surgical options include thrombectomy and phlebectomy, entailing the removal of the thrombus or the affected vein, respectively, as well as ligation of the affected vein. These procedures traditionally served patients with extensive SVT or recurrent symptoms.^5^

In the past, emergency or urgent surgical therapy involving the ligation of the SFJ or saphenopopliteal junction (SPJ) was the standard approach for rapidly ascending or orifice-related SVT of the superficial truncal veins to prevent DVT by ingrowth into the deep venous system. However, a paradigm shift in this approach towards conservative treatment has emerged in recent years.^3^

Despite the presence of substantial evidence and guidance, the management of SVT exhibits considerable variability.^6^^,^^7^ For instance, the ESVS guideline recommends treatment of superficial reflux after the acute phase of SVT, typically no sooner than 3 months following onset.^3^ This wide-ranging variation in real-world SVT management and outcomes has been the subject of several observational studies. The InNvestigating SIGnificant Health TrendS in the management of Superficial Vein Thrombosis (INSIGHTS-SVT) study has systematically collected comprehensive data on patient characteristics, diagnosis, management, and outcomes of acute isolated SVT in Germany under real-world conditions.

In this report, we present an analysis of the characteristics and outcomes of patients who underwent invasive interventions for the initial SVT episode during the period between their study enrollment and the 12-month follow-up visit.

## Methods

In brief, INSIGHTS-SVT was a prospective, multi-center, observational study spanning a 12-month follow-up period. The study protocol received approval by the institutional review board of the physician chamber in Hessia, Germany, and all patients provided written informed consent. The study was registered by the regulatory authority (BfArM) under NIS 6781 and by ClinicalTrials.gov under NCT 02,699,151.^8^ Previous reports have covered the 3-month^9^ and 12-month outcomes of the entire patient cohort, as well as the outcomes specific to patients with cancer.^10^

The study engaged hospital- and office-based physicians with expertise in the treatment of SVT, who were certified in compression ultrasound (CUS) diagnostics. This group included vascular physicians, vascular surgeons, phlebologists, and general internal medical physicians or general practitioners.

Patient inclusion criteria were as follows: objectively confirmed (by CUS, including duplex ultrasound [DUS]), time interval between onset of SVT symptoms and inclusion less than 3 weeks, isolated SVT of the lower extremities; concomitant DVT was excluded by CUS or DUS.

Patients were ineligible if they met any of the following exclusion criteria: proximal extension of SVT to 3 cm or less of the SFJ, symptoms suggestive of PE; subjects unlikely to comply with the requirements of the protocol (eg, due to cognitive and/or language limitations); and subjects likely not available for 12-month follow-up. Patients had a follow-up visit at 3 months and at 12 months; optional visits were at 10 ± 3 days and 45 ± 3 days, respectively. Due to the observational nature of the study, ultrasound examinations and any other diagnostic or therapeutic decisions during follow-up were at the investigators’ discretion. DUS refers to duplex ultrasound systems with both pulsed-wave Doppler and color technology.

The primary outcome measure was the incidence of symptomatic VTE, defined as a composite of DVT, PE, and recurrent or extending SVT at 3 months of follow-up. Secondary outcomes included recurrent SVT or extension of SVT into the deep venous system or to 3 cm or less from the SFJ, symptomatic PE, DVT, persistent SVT (clinical non-improvement), asymptomatic SVT, death, new cancer, or cancer relapse and hospitalization because of VTE, over 12 months.

The primary safety outcome measure was the combination of major or clinically relevant non-major bleeding at 3 months, with definitions based on American College of Chest Physicians Evidence-Based Clinical Practice Guidelines (major bleeding)^11^ and the CALISTO trial (clinically relevant non-major bleeding).^12^

Information pertaining to invasive procedures was systematically recorded using a predefined list of interventions, which encompassed the following: sclerotherapy, endovenous thermal ablation (EVTA), ligation of the SFJ or SPJ, vein stripping, thrombectomy, phlebectomy, and any other relevant procedures. Further, information on pharmacological and non-pharmacological therapy, including type of utilized drugs, their dosing, and duration of application was collected.

### Statistical analysis

In this pre-defined subgroup analysis, we conducted a comparison between patients who received invasive interventions within the 12-month follow-up period and those who did not. We utilized standard descriptive statistics to present the distribution of selected parameters. Patient comparisons were carried out using analysis of variance and χ^2^ tests as appropriate.

To investigate the incidence of the VTE outcome over the 3-month and 12-month follow-up period, we employed Kaplan-Meier estimates. To identify potential correlates of VTE in the follow-up period, both univariate and multivariate Cox proportional hazard models were applied. We assessed the proportional hazard assumption by examining Schoenfeld residuals.

The incidence rate (based on observation time) of VTE outcome was calculated in patients without any invasive interventions and patients with VTE event before invasive intervention in comparison to patients with VTE outcome after invasive interventions. The incidence rate ratio for the two groups was estimated by multilevel mixed-effects Poisson regression analysis.

Our analysis of the VTE outcome considered cumulative data within the baseline to 12-month time frame and the 3-month to 12-month follow-up window. The threshold for statistical significance was set at a *P*-value of .05.

All statistical analyses were conducted using Stata Statistical Software, Release 12.1 (StataCorp LLC).

## Results

**Fig 1:**
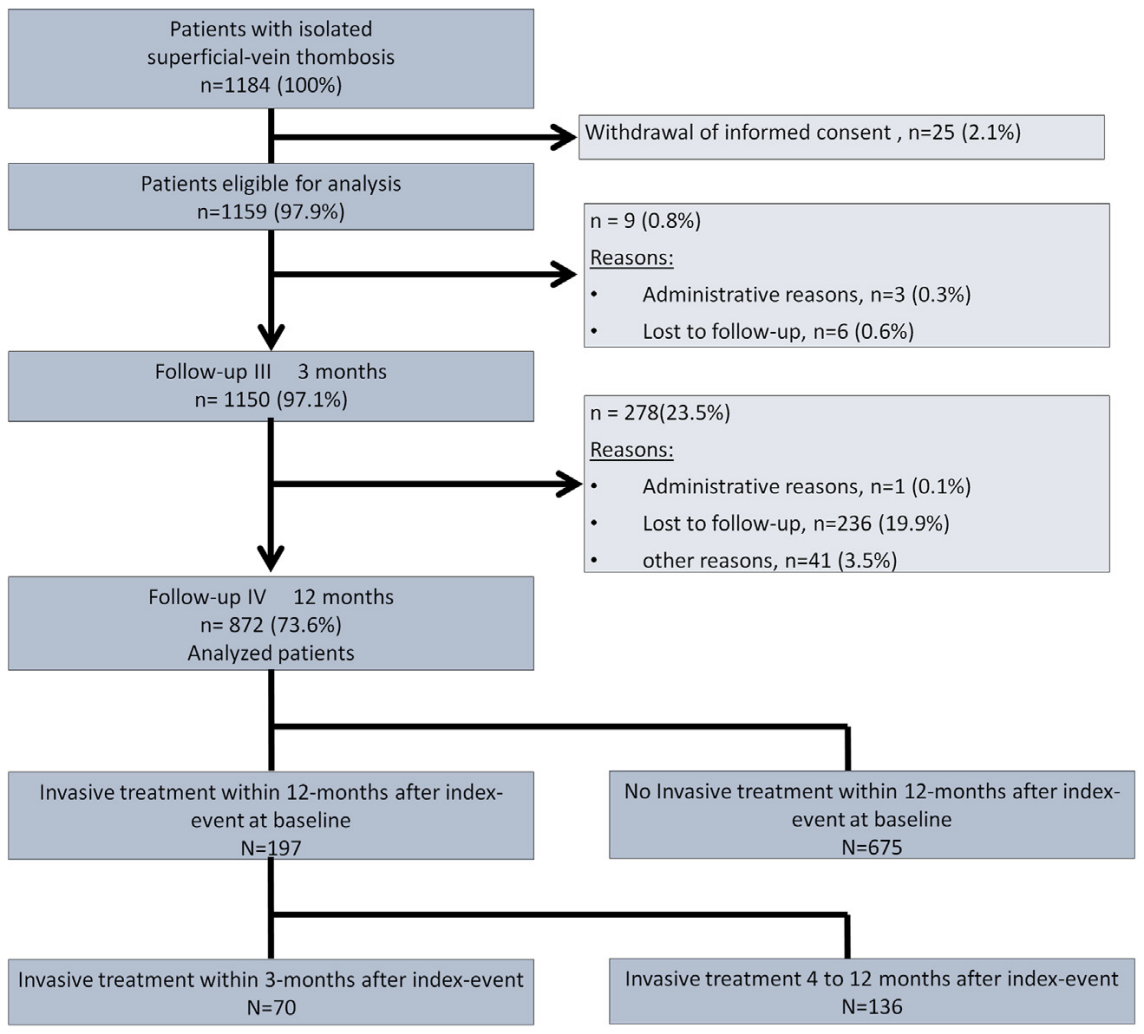
Study flowchart. In addition, there were nine patients who received invasive treatment twice, spanning both periods.

The study flow is illustrated in Fig 1. Of the 872 patients with available 12-month follow-up data, 197 patients (22.6%) underwent invasive treatments at some time point during the follow-up. Among these 197 patients receiving invasive treatments, 70 received such interventions within the first 3 months, 136 patients between 4 and 12 months, and nine patients received invasive treatment twice, spanning both periods.

A total of 817 patients (93.4%) received anticoagulation therapy after the index event as the initial treatment choice at baseline. Of the 197 patients, 182 patients (92.4%) undergoing surgery and 635 of the 675 patients (94.1%) not undergoing surgery received anticoagulation. Furthermore, 141 of 197 patients (71.6%) received some form of non-pharmacological treatment in addition to the anticoagulation therapy. The median duration of anticoagulation therapy was 28 days (interquartile range [IQR], 18-42 days) for patients who underwent surgery and 35 days (IQR, 21-42 days) for those who did not.

Table I displays a comparison of the characteristics between the 197 patients who underwent invasive treatments and the 675 patients who received conservative treatment. All 197 patients who underwent invasive treatments had SVT as the index event in a varicose vein. No significant differences were observed in age, sex, body mass index, or ethnicity between the two groups. There were noteworthy distinctions in chronic risk factors for VTE. Invasively treated patients more frequently presented with varicose veins and chronic venous insufficiency/ulceration but had a lower incidence of prior thrombosis. No other factors showed statistically significant differences.

**Table I:** Patient characteristics at inclusion

	Conservatively treated		Invasively treated	*P* value^b^
	Varicose veins	No varicose veins		
	(n = 520)	(n = 155)	(n = 197)	
	No. (%)	No. (%)	No. (%)	
Age, years	62.3 (14.4)	58.3 (15.6)	58.1 (13.2)	**<.001**
Age ≥65 years	253 (48.7)	55 (35.5)	62 (31.5)	**<.001**
Women	341 (65.6)	98 (63.2)	123 (62.4)	.691
Body mass index, kg/m2	29.5 (6.5)	29.2 (6.7)	29.1 (5.6)	.733
Body mass index ≥30 kg/m2	206 (39.6)	60 (38.7)	69 (35.0)	.528
Caucasian	517 (99.4)	155 (100.0)	197 (100.0)	.361
Chronic risk factors for VTE				
Varicose veins	416 (80.0)	76 (49.0)	174 (88.3)	**<.001**
History of thrombosis				
SVT	181 (34.8)	38 (24.5)	59 (30.0)	**.044**
DVT or PE	103 (19.8)	33 (21.3)	12 (6.1)	**<.001**
VTE (SVT, DVT, or PE)	242 (46.5)	57 (36.8)	66 (33.5)	**.003**
Family history of DVT or PE	80 (15.4)	29 (18.7)	36 (18.3)	.484
CVI/ulceration	271 (52.1)	52 (33.6)	111 (56.4)	**<.001**
Cancer	50 (9.6)	5 (3.2)	8 (4.1)	**.004**
Known thrombophilia	29 (5.6)	15 (9.7)	6 (3.1)	**.028**
Hormone replacement therapy	11 (2.1)	1 (0.7)	2 (1.0)	.334
Oral contraception	37 (10.9)	18 (18.4)	10 (8.1)	**.049**
Current smoking	64 (12.3)	33 (21.3)	39 (19.8)	**.005**
Hemiplegia	4 (0.8)	0 (0.0)	0 (0.0)	.218
Chronic inflammatory disease	27 (5.2)	8 (5.2)	7 (3.6)	.642
Immobility/bedriddenness	25 (4.8)	4 (2.6)	6 (3.1)	.340
Arterial risk factors^a^	313 (60.2)	79 (51.0)	84 (42.6)	**<.001**
Heart failure	16 (3.1)	6 (3.9)	0 (0.0)	.321
Respiratory failure	27 (5.2)	4 (2.6)	2 (1.0)	**.022**
Transient risk factors				
Trauma (past 4 weeks)	19 (3.7)	10 (6.5)	7 (3.6)	.276
Travel (>6 hours by car or flight)	56 (10.8)	12 (7.7)	11 (5.6)	.080
Major surgery (past 12 weeks)	21 (4.0)	4 (2.6)	10 (5.1)	.496
Severe systemic infection	7 (1.4)	1 (0.7)	0 (0.0)	.223
Pregnancy	4 (0.8)	1 (0.7)	2 (1.0)	.920
Postpartum	4 (0.8)	3 (1.9)	2 (1.0)	.451
Characteristics of SVT events				
Great or lesser saphenous vein	262 (50.4)	127 (81.9)	92 (46.7)	**<.001**
Other veins	258 (49.6)	28 (18.1)	105 (53.3)	
Great saphenous vein only	177 (34.0)	91 (58.7)	60 (30.5)	**<.001**
Distance between thrombus and SFJ, cm	26.2 (15.4)	26.1 (14.8)	26.7 (14.2)	.965
Distance between thrombus and SFJ <10 cm	25 (13.7)	8 (8.6)	8 (11.8)	.471
Lesser saphenous vein only	30 (5.8)	8 (5.2)	10 (5.1)	.916
Number of affected veins	2.1 (1.0)	2.7 (1.6)	2.2 (0.8)	.424
Localization				
Proximal only	128 (24.8)	36 (25.2)	47 (24.2)	.885
Distal only	290 (56.1)	75 (52.5)	105 (54.1)	
Proximal and distal	99 (19.2)	32 (22.4)	42 (21.7)	
Extension				
Mean (SD)	13.8 (11.0)	17.7 (11.7)	13.2 (9.5)	**<.001**
<20 cm	360 (69.5)	88 (57.1)	141 (71.6)	**.007**
≥20 cm	158 (30.5)	66 (42.9)	56 (28.4)	

*CVI*, Chronic venous insufficiency; *DVT*, deep vein thrombosis; *PE*, pulmonary embolism; *SD*, standard deviation; *SFJ*, saphenofemoral junction; *SVT*, superficial vein thrombosis; *VTE*, venous thromboembolism.

Data are presented as number (%) or mean (standard deviation).

Boldface *P* values indicate statistical significance.

a Diabetes mellitus, arterial hypertension, coronary artery disease, cerebrovascular disease, peripheral artery disease, atrial fibrillation, renal failure.

b *P* value for the comparison of patients with any intervention vs conservatively treated patients.

**Fig 2:**
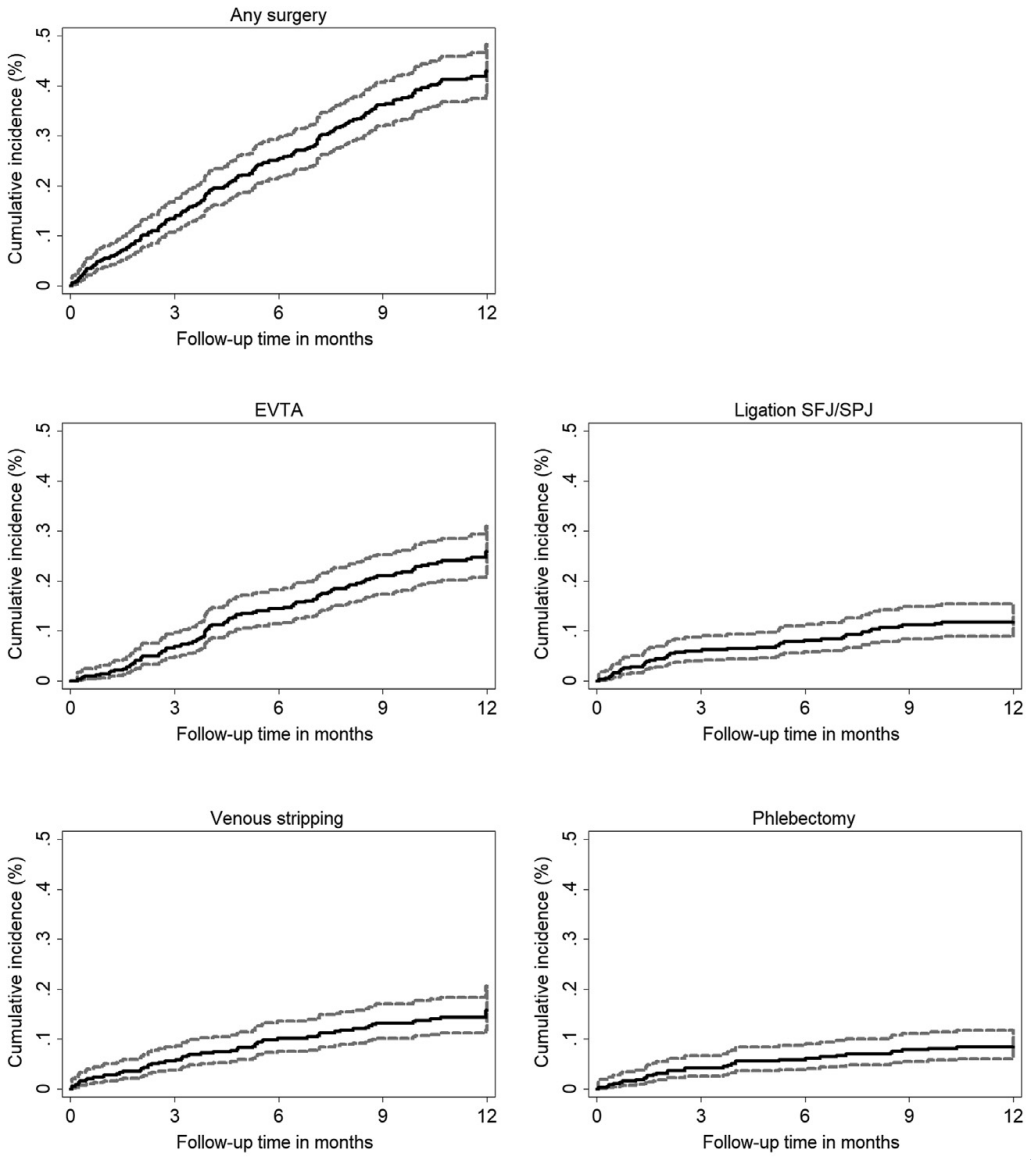
Lines represent cumulative event rates in percent, along with their 95% confidence intervals (CIs) over time until 12 months, categorized by type of intervention. *EVTA*, Endovenous thermal ablation; *SFJ/SPJ*, ligation of the saphenofemoral or saphenopopliteal junction.

Regarding specific procedures, the most commonly performed interventions within the first 3 months included EVTA (3.6%), vein stripping (2.9%), and ligation of the SFJ/SPJ (2.8%). During the acute phase, which spanned up to 1 week, only five patients underwent surgery. In the subsequent 1 to 4 weeks, 19 patients received surgical interventions, whereas between 4 and 12 weeks, 46 patients underwent surgery, and between 12-week and 1-year follow-up, 136 underwent surgery. Further details on the types of interventions and surgical procedures carried out in patients with SVT, categorized by the time interval between diagnosis and intervention, are shown in Fig 2 (including Kaplan-Meier curve of surgery combined).

A great majority of patients enrolled in the registry (93.4%) received anticoagulation at baseline. Notably, concerning both anticoagulant and non-pharmacological treatments, the differences between patients who underwent surgery and those who did not were minimal and did not reach statistical significance. Specifically, among patients who received surgery compared with those who did not, fondaparinux was administered at baseline to 125 patients (63.5%) vs 417 (61.8%), low molecular weight heparins were given to 48 patients (24.4%) vs 170 (25.2%), and other anticoagulants (direct oral anticoagulants, vitamin K anticoagulants, and unfractionated heparin) were used by nine patients (4.6%) vs 48 (6.7%). Additionally, non-pharmacological treatment was utilized by 141 patients (71.6%) who underwent surgery.

The primary outcome of symptomatic VTE within the initial 3 months of follow-up was observed in 51 patients (7.6%) treated conservatively, which included 37 patients with varicose veins and 14 patients without varicose veins. In contrast, 16 patients (8.1%) who underwent surgical treatment experienced this outcome. Importantly, this data indicates that there was no significant difference between the two groups in terms of symptomatic VTE within the first 3 months. Furthermore, even in the subgroup analysis categorized by the type of invasive therapy, no significant differences were identified in the endpoints compared with conservatively treated patients after the initial 3 months of follow-up.

Furthermore, there was no statistically significant difference in the incidence of symptomatic VTE within the 12-month follow-up period. Specifically, among conservatively treated patients, those with varicose veins (n = 65; 12.5%) and those without varicose veins (n = 14; 11.0%) exhibited similar rates, as did invasively treated patients (n = 26; 13.2%).

**Fig 3:**
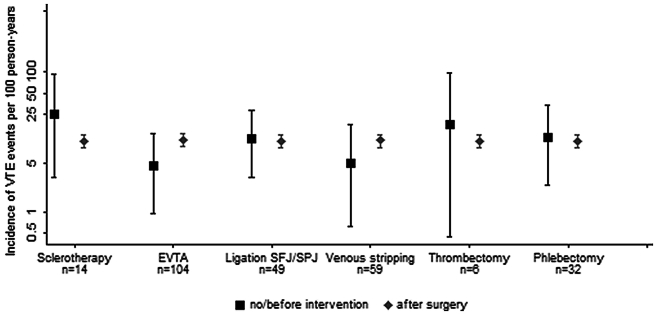
The figure shows incidence rates (IRs) of venous thromboembolism events (*VTEs*) per 100 patient-years, by intervention type. The *black squares* show VTE incidence rates along with 95% confidence intervals (CIs) for patients with no intervention or before intervention, whereas the *gray diamonds* show VTE IRs with 95% CIs for patients after intervention over 12 months. IRs for the different interventions: Sclerotherapy: IR, 2.09; 95% CI, 0.52-8.50; *P* = .302; endovenous thermal ablation (*EVTA*): IR, 0.37; 95% CI, 0.12-1.18; *P* = .092; ligation of the saphenofemoral or saphenopopliteal junction (*SFJ/SPJ*): IR, 1.03; 95% CI, 0.38-2.91; *P* = .952; vein stripping: IR, 0.44; 95% CI, 0.11-1.78; *P* = .248; thrombectomy: IR, 1.77; 95% CI, 0.25-12.72; *P* = .570; phlebectomy: IR, 1.03; 95% CI, 0.33-3.25; *P* = .960.

An additional analysis focused on the incidence of VTE outcome before and after invasive therapy as well as in conservatively treated patients. A numerically lower incidence of VTE was observed for patients after EVTA as compared with conservatively treated patients and to patients before EVTA (4.6 vs 10.9 events per 100 person-years). Venous stripping was also associated with a lower incidence of VTE outcome after invasive intervention (5.1 vs 10.5 events per 100 person-years). The differences between the two groups were not statistically significant (Fig 3).

Table II (within 3 months) and Table III (within 12 months) present the incidence of VTE and bleeding events for conservatively treated patients with and without varicose veins in comparison to surgically treated patients. Notably, patients who underwent EVTA or sclerotherapy did not exhibit a statistically significant difference in the incidence of symptomatic VTE compared with conservatively treated patients.

**Table II:** Three-month incidence of venous thromboembolic and bleeding events

	Conservatively treated with varicose veins (n = 520)		Conservatively treated without varicose veins (n = 155)		Invasively treated (n = 197)	
Primary outcome						
Symptomatic VTE (DVT, PE, recurrent or extending^b^ SVT)	37	7.1	14	9.0	16	8.1
Secondary outcome						
SVT (recurrent or extending^a^)	30	5.8	9	5.8	15	7.6
PE	4	0.8	4	2.6	1	0.5
DVT	10	1.9	2	1.3	1	0.5
DVT and PE	12	2.3	6	3.9	1	0.5
Persistent SVT	34	6.5	5	3.2	2	1.0
Asymptomatic SVT^b^	1	0.2	1	0.7	0	0.0
Death	2	0.4	1	0.7	0	0.0
New cancer or relapse	8	1.5	1	0.7	0	0.0
Hospitalization due to VTE	6	1.2	1	0.7	0	0.0
Bleeding	8	1.5	6	3.9	3	1.5
Severe bleeding	2	0.4	1	0.7	0	0.0
Clinically relevant non-major bleeding	6	1.2	5	3.2	3	1.5

*DVT*, Deep vein thrombosis; *PE*, pulmonary embolism; *SFJ*, saphenofemoral junction; *SVT*, superficial vein thrombosis; *VTE*, venous thromboembolism.

a Extension into the deep vein system or <3 cm to the SFJ).

b Detectable only on compression ultrasound or duplex ultrasound.

**Table III:** Twelve-month incidence of venous thromboembolic and bleeding events

	Conservatively treated with varicose veins (n = 520)		Conservatively treated without varicose veins (n = 155)		Invasively treated (n = 197)	
Primary outcome						
Symptomatic VTE (DVT, PE, recurrent or extending^a^ SVT)	65	12.5	17	11.0	26	13.2
Secondary outcome						
SVT (recurrent or extending^a^)	50	9.6	10	6.5	23	11.7
PE	8	1.5	4	2.6	2	1.0
DVT	17	3.3	4	2.6	2	1.0
DVT and PE	21	4.0	8	5.2	3	1.5
Persistent SVT	35	6.7	6	3.9	2	1.0
Asymptomatic SVT^b^	3	0.6	1	0.7	1	0.5
Death	8	1.5	3	1.9	1	0.5
New cancer or relapse	9	1.7	1	0.7	1	0.5
Hospitalization due to VTE	7	1.4	1	0.7	0	0.0
Bleeding	9	1.7	6	3.9	3	1.5
Severe bleeding	2	0.4	1	0.7	0	0.0
Clinically relevant, non-major bleeding	7	1.4	5	3.2	3	1.5

*DVT*, Deep vein thrombosis; *PE*, pulmonary embolism; *SFJ*, saphenofemoral junction; *SVT*, superficial vein thrombosis; *VTE*, venous thromboembolism.

a Extension into the deep vein system or <3 cm to the SFJ).

b Detectable only on compression ultrasound or duplex ultrasound.

Among surgically treated patients, symptomatic VTE was observed in 20 patients (74%) before the intervention and in seven patients (26%) after the intervention (*P* = .021).

Detailed information on the performed surgical interventions is provided in Table IV. Among all patients who received invasive treatments, one-third (n = 70; 35.5%) underwent surgery within 3 months of SVT diagnosis, whereas two-thirds (n = 136; 64.5%) received surgery more than 3 months after SVT diagnosis. The most common surgical procedures included EVTA (n = 106; 12.1%), followed by SFJ/SPJ ligation (n = 52; 6.0%), and venous stripping (n = 51; 5.8%).

**Table IV:** Surgical interventions by type and time period

	Total (within 3 months after diagnosis)		Within 1 week after diagnosis		2-4 weeks after diagnosis		>4-12 weeks after diagnosis		More than 12 weeks after diagnosis	
	(n = 872)		(n = 872)		(n = 872)		(n = 872)		(n = 872)	
Any surgery	70	8.0	5	0.6	19	2.2	46	5.3	136	15.6
Sclerotherapy	2	0.2	0	0.0	0	0.0	2	0.2	12	1.4
EVTA	31	3.6	1	0.1	5	0.6	25	2.9	75	8.6
Ligation SFJ/SPJ	24	2.8	1	0.1	9	1.0	14	1.6	28	3.2
Venous stripping	25	2.9	3	0.3	6	0.7	16	1.8	36	4.1
Thrombectomy	6	0.7	1	0.1	3	0.3	2	0.2	0	0.0
Phlebectomy	16	1.8	1	0.1	5	0.6	10	1.2	15	1.7
Other	9	1.0	0	0.0	4	0.5	5	0.6	5	0.6
Isolated ligation SFJ/SPJ	7	0.8	1	0.1	4	0.5	3	0.3	11	1.3
Ligation SFJ/SPJ in combination with thrombectomy	6	0.7	0	0.0	3	0.3	2	0.2	0	0.0
Ligation SFJ/SPJ, (non-isolated) phlebectomy and stripping	4	0.5	0	0.0	1	0.1	3	0.3	1	0.1
Isolated phlebectomy	0	0.0	0	0.0	0	0.0	1	0.1	3	0.3

*EVTA*, Endovenous thermal ablation; *SFJ/SPJ*, saphenofemoral/saphenopopliteal junction.

## Discussion

The findings of this study shed light on the utilization of invasive treatments in the management of SVT, revealing that such procedures were relatively infrequent, accounting for only 8.0% of all patients within the first 3 months after study inclusion or 23.6% during the complete 12-month follow-up. Overall, the group of patients treated invasively appeared to be “low-risk,” as they were younger, had fewer previous VTE events, but had a higher rate of varicose veins in their medical history.

Almost all patients in the study received anticoagulation alone or in combination with surgical interventions. The rates of anticoagulation therapy, as well as the duration of such treatment, were similar between the groups treated surgically and those treated conservatively. Therefore, we conclude that anticoagulation therapy did not significantly confound the results of our study.

Among the spectrum of interventions, EVTA, venous stripping, and ligation of the SFJ/SPJ emerged as the most commonly performed procedures, with the majority being performed after a 12-week period following SVT diagnosis. The study lacks detailed information regarding the rationale behind the early surgical interventions. Nonetheless, for patients undergoing surgery at a later stage, varicose veins in conjunction with a history of SVT consistently served as the primary indication. In summary, the study showed that early surgical intervention of superficial phlebitis was associated with low risk for VTE complications. The study’s key finding suggests that there is no significant difference in the incidence of symptomatic VTE between conservatively and invasively treated patients with acute isolated SVT of the lower limbs. Additionally, there were also no statistically significant differences observed in bleeding events between the treatment groups. However, it is crucial to underscore that invasive treatment may inherently heighten the risk of bleeding events. Consequently, the decision regarding treatment modality should be based on a careful individual assessment of the potential risks and benefits for each patient.

Moreover, the results imply a potential protective effect against VTE events when applied after 3 months from the index SVT event. Our finding is in line with the German DVT guidelines, where interventions are recommended not earlier than 3 months after SVT symptoms have decreased.

These outcomes align with previous studies, including a Cochrane systematic review by Di Nisio et al,^4^ which concluded that anticoagulation alone typically suffices for SVT treatment, and the evidence for surgical interventions is not compelling. The ESVS 2021 Clinical Practice Guidelines on the Management of Venous Thrombosis also advocate for anticoagulation as the primary approach for SVT treatment, specifically recommending surgical or endovascular interventions only after the inflammatory and prothrombotic phase has concluded, which is typically no sooner than 3 months after SVT onset.³ This guideline reflects a common clinical practice, although it is not primarily based on robust evidence.

However, it is important to note that these guidelines acknowledge certain circumstances in which surgical interventions may be warranted, such as when the thrombus extends into the deep venous system, when complications like ulceration or bleeding arise, or when anticoagulation is not possible. Additionally, the 2023 guideline on the Diagnosis and Therapy of Deep Vein Thrombosis by the German Society of Angiology emphasizes the potential advantages of surgical interventions in specific situations, such as reducing the risk of recurrent thrombosis and alleviating symptoms. These guidelines collectively underscore the importance of a tailored and nuanced approach to SVT management, considering both individual patient characteristics and the clinical context.^5^

The strengths and limitations of the study are duly acknowledged and provide valuable context for the interpretation of the results: Strengths include the availability of a large cohort from clinical practice with relatively long follow-up, documentation of patients in experienced clinical centers, consecutive enrollment of eligible patients, and the very broad inclusion criteria. Thus, this prospective real-life registry provides a major advantage in that it reflects the full spectrum of everyday practice and current treatment pathways, which would not be possible in a randomized controlled trial.

Limitations include the open non-randomized study design, with the decision to perform surgical interventions being at the discretion of the treating physician. This introduces a heterogenous study sample with potential selection bias, as some patients who could have benefited from surgery may not have been selected for intervention. Conversely, it is also possible that patients with more severe varicose veins were more likely to be selected for surgery, potentially leading to an overestimation of the effect. Also, the study lacks detailed information about the specific indications for interventions, such as the extent of the SVT, or the severity of the symptoms. The combined outcome of VTE is mostly driven by superficial thrombophlebitis events but DVT and PE are most of interest for patient-centerd outcomes. Understanding these indications could provide more insight into the decision-making process.

Patients with SVT located less than 3 cm from the SFJ or SPJ were excluded from the registry. This exclusion may have omitted a group of patients who may have been suitable candidates for intervention, which may affect the generalizability of the study. The study had a relatively small sample size, especially in the group of patients treated invasively. This limited sample size may affect the statistical power of the analysis. The study primarily assessed short-term outcomes within 3 and 12 months of diagnosis. Therefore, it did not evaluate long-term outcomes or quality of life, which may be important factors in the decision-making process for SVT management. Long-term follow-up studies may be needed to determine the potential long-term benefits or risks of surgical intervention.

## Conclusions

In conclusion, this study, with real-world data, contributes to our understanding of SVT management by highlighting the importance of individualized treatment strategies, appropriate timing of interventions, and the need for continued research to refine our approach to this condition. It reinforces the notion that the management of SVT should be guided by a careful assessment of each patient’s individual circumstances and risk factors.

## Author Contributions

Conception and design: TN, RR, UH, AS, AH, FL, DP, JK, HG, RB

Analysis and interpretation: TN, RR, UH, AS, AH, FL, DP, JK, HG, RB

Data collection: DP

Writing the article: TN, DP, JK, RB

Critical revision of the article: TN, RR, UH, AS, AH, FL, DP, JK, HG, RB

Final approval of the article: TN, RR, UH, AS, AH, FL, DP, JK, HG, RB

Statistical analysis: JK

Obtained funding: AS, AH, RB

Overall responsibility: TN

## Disclosures

E.R. has received honoraria for lectures and advisory boards from Bayer, Boehringer Ingelheim, Daiichi-Sankyo, Leo Pharma, and Pfizer. R.B. has received research support from AFNET, CPC, and FADOI; and honoraria from Bayer, BMS, Leo, Pfizer, and Viatris. H.G. has received honoraria for lectures and advisory boards from Aspen, Mylan, Bayer, and Boehringer-Ingelheim. D.P. has received honoraria for consultancy, advisory boards, or lectures by Actelion, Bayer, Biogen, Aspen, Amgen, MSD, Boehringer Ingelheim, Novartis, Daiichi Sankyo, Genzyme, and Zambon. F.L. has received honoraria for lectures or consultancy from Aspen, Bayer, Bristol-Myers Squibb, Daiichi Sankyo, LEO Pharma, Pfizer, Sanofi, and Viatris. U.H. has received research support and honoraria for lectures and advisory boards from Bayer HealthCare Pharmaceuticals, Bristol-Myers-Squibb, Pfizer, Boehringer Ingelheim, Daiichi Sankyo, Leo Pharma, and Aspen. T.N. has received honoraria for consultancy from Medi Bayreuth; and honoraria for presentations from Aspen, Bayer, Bristol-Myers Squibb, and Mylan. A.H. was, at the time of the study, a full-time employee of Aspen Pharma GmbH, Munich, and is now an employee of Amgen GmbH, Germany. A.S. is a full-time employee of Mylan Germany GmbH, Germany.

## Figures and Tables

**Supplementary Table:** Investigator List: The following investigators/sites documented patients (collaborators):

Dr. Christian. Schnabl, Berlin
Dr. Tina. Winterbauer, Birkenau
Dr. Norbert. Schön, Mühldorf
Harriet Simone. Werno, Nürnberg
Dr. Georg. Herman, Augsburg
PD Dr. Oliver. Schmidt, Erlangen
Dr. Beate. Dietrich, Gera
Dr. Martin, Schünemann, Nörten-Hardenberg
Dr. Eberhard. Rieker, Lauffen
Dr. Ulrich. Ruppe, Berlin
Dr. Gabriele. Betzl, München
Dr. Thomas. Noppeney, Nürnberg
Dr. Peter. Heilberger, Nürnberg
Dr. Dimitrios. Tsantilas, Augsburg
Dr. Andreas. Köpp, Braunschweig
Dipl med. Lutz. Forkmann, Chemnitz
Dr. Andreas. Willeke, Darmstadt
Dr. Gabriele. Rothenbücher, Dornstadt
Dr. Karl. Förster, Ehringshausen
Dr. Jeanette. Kießling, Erfurt
Dr. Gesche. Junge, Heidelberg
Dr. Ina. Wittig,Leipzig
Dr. Dagmar. Wilms, Leverkusen
Dr. Christoph. Schulte, Limburg
Dr. Stephan. Flüchter, Mannheim
Dr. Martina. Kneist, Mühlhausen
Dr. Ulrike. Kirsch, Oranienburg
Dr. Thomas. Herrmann, Weinheim
Alexandra. Turowski, Berlin
Dr. Karsten. Hartmann, Freiburg
Dr. Wolfram. Oettler, Görlitz
Dr. Heike. Nelles, Altenburg
Dr. Jürgen. Frank, Baesweiler
Dr. Savvas. Apostolidis, Frankfurt/Main
Dr. Dag-Alexander. Keilhau, Hamburg
Dr. Renate. Murena Schmidt, Köln
Dr. Iris. Rocha Rivera-Reuver, Köln
Dr. Kerstin. Augustin, Mosbach
Diethard. Predel, Nordhausen
Dr. Thomas. Hertel, Zwickau
Dr. Ursula. Schmeink, Aachen
Dr. Simone. Seibt, Berlin
Dr. Jürgen. Schreiner, Dachau
Dr. Christine. Zollmann, Jena
Dr. Eckart. Möbius, Schwerin
Dr. Thomas. Vollmer, Bruchsal
Dr. Roswitha. Brettschneider, Düren
Dr. Sabine. Raulin, Karlsruhe
Dr. Siamak. Pourhassan, Oberhausen
Dr. Gerlind. Läger, Oelsnitz
Dr. Robert. Brandl, Passau
Dr. Rainer. Schmiedel, Kaiserslautern
Karoline. Jager, Saarbrücken
Dr. Erika. Mendoza, Wunstorf
Jörg. Schwuchow, Neuruppin
Dr. Jan-Peter. Siegers, Otterndorf
Peter. Gätzschmann, Viechtach
Dr. Dimitrios. Zgouras, Wiesbaden
Prof Dr. Werner. Lang, Erlangen
Arne. Clasing, Lüneburg
Dr. Anatoli. Ananin, Nürnberg
Dr. Jörg. Rutkowski, Traunstein
PD Dr. Christoph. Kalka, Brühl
Dipl med. Frank. Ackermann, Greiz
Dr. Fred. Peter, Bamberg
Dr. Patricia. Schaub, Limburg
Dr. Jan. Beyer-Westendorf, Dresden
Dr. Bernadette. Brado, Heidelberg
Dr. Mario. Schöniger, Nordendorf
Dr. Sven. Köpnick, Wuppertal
Dr. Ferenc. Biro, Köln
Prof. Birgit. Linnemann, Frankfurt am Main

## Appendix

The authors would like to thank Maria Kretschmer and Linda Kottke of GWT-TUD GmbH, Dresden, for their efficient project management.
